# Evaluation of Fine Aggregate Morphology by Image Method and Its Effect on Skid-Resistance of Micro-Surfacing

**DOI:** 10.3390/ma11060920

**Published:** 2018-05-29

**Authors:** Yue Xiao, Feng Wang, Peide Cui, Lei Lei, Juntao Lin, Mingwei Yi

**Affiliations:** 1 State Key Laboratory of Silicate Materials for Architectures, Wuhan University of Technology, Wuhan 430070, China; xiaoy@whut.edu.cn (Y.X.); wangfengfeng@whut.edu.cn (F.W.); 2 Shenzhen MacRitchie Technology Co., Ltd., Shen Zhen 518101, China; nm3rnd@gmail.com; 3 Faculty of Engineering, China University of Geosciences (Wuhan), Wuhan 430074, China; linjt@cug.edu.cn; 4 National Engineering Research Center of Road Maintenance Technologies, Beijing 100095, China; cwhymw@gmail.com; 5 Research Institute of Highway Ministry of Transport, Beijing 100088, China

**Keywords:** aggregate morphology, aggregate image measurement system, micro-surfacing, skid-resistance, surface texture

## Abstract

Micro-surfacing is a widely used pavement preventive maintenance technology used all over the world, due to its advantages of fast construction, low maintenance cost, good waterproofness, and skid-resistance performance. This study evaluated the fine aggregate morphology and surface texture of micro-surfacing by AIMS (aggregate image measurement system), and explored the effect of aggregate morphology on skid-resistance of single-grade micro-surfacing. Sand patch test and British pendulum test were also used to detect skid-resistance for comparison with the image-based method. Wet abrasion test was used to measure skid-resistance durability for feasibility verification of single-grade micro-surfacing. The results show that the effect of Form2D on the skid-resistance of micro-surfacing is much stronger than that of angularity. Combining the feasibility analysis of durability and skid-resistance, 1.18–2.36 grade micro-surfacing meets the requirements of durability and skid-resistance at the same time. This study also determined that, compared with British pendulum test, the texture result obtained by sand patch test fits better with results of image method.

## 1. Introduction

With the development of transportation industry, the construction of the world’s highway networks has gradually improved. The maintenance and disease prevention of asphalt pavements have already attracted widespread attention. Micro-surfacing is a widely used pavement preventive maintenance technology used all over the world, due to its advantages of fast construction, low maintenance cost, good waterproofness, and skid-resistance performance [1,2,3,4]. It consists of modified emulsified asphalt, aggregates, fillers, water, and additives. The number of traffic accident can be reduced if there is enough friction between tire and pavement surface [5,6]. Safe and comfortable conditions of micro-surfacing are directly related to abundant surface texture characteristics, which makes great contributes to skid-resistance [7,8,9].

Evaluation of asphalt pavement texture mainly includes microtexture and macrotexture. Macrotexture is closely related to the distribution and gradation of aggregates, while microtexture is dependent on aggregate morphological characteristics [10,11,12,13]. The traditional test methods for texture property of asphalt pavements mainly include sand patch test and British pendulum test. These traditional testing methods are not only time-consuming, but also depend largely on the operator’s subjective judgment, making the experimental results imprecise and incomplete. Accurate quantification of texture characteristics has great significance to fully understanding the skid-resistance of pavement.

Digital image processing technology has been used for accurately quantifying the texture characteristics of pavement, and a variety of equipment and image testing methods based on digital image processing technology has been rapidly developed [14,15,16,17]. AIMS (aggregate image measurement system) is an objective and credible instrument for evaluating morphological characteristics of aggregates and surface texture of pavements, which allows accurate and comprehensive analysis, and calculation of texture based on digital images collected by a high resolution digital camera and a variable magnification microscope [18,19,20,21].

Asi et al. performed a comparative study to find the British pendulum skid-resistance number for a number of mixes. It was found that the mix with 30% slag has the highest skid number, followed by Superpave, SMA, and Marshall mixes, respectively. Also, increasing the asphalt content above the optimal asphalt content value decreases the skid-resistance of these mixes [7]. Puzzo et al. presented an image-based technique for the assessment of a 3D model of pavement texture to calculate the digital mean texture depth (MTD), starting from the digital surface model (DSM) generated by the photos [22]. Araujo et al. detected characterization of three hot mix asphalt (HMA) texture properties using digital image processing techniques, and the results indicated that the HMA particle size distribution directly affects its texture characteristics [15]. Chen et al. used fractal dimension of the fracture surface to evaluate impact of contact stress distribution on skid-resistance of asphalt pavements. The result showed that the stress distribution increased with an increase of pavement texture depth or tire load, or a decrease of the tire inflation pressure. The influence of pavement roughness and tire load was more significant than tire inflation pressure on the stress concentration [23]. El-Desouky used a special fluid to conduct skid-resistance tests at below freezing temperatures, and test results were used to develop correction models for skid-resistance under temperature variations [24]. Although many studies focus on skid-resistance of asphalt pavements, no research has been done to correlate skid-resistance of micro-surfacing with fine aggregate morphologies.

The objective of this study is to evaluate fine aggregate morphology and surface texture of micro-surfacing by AIMS, and explore the effect of aggregate morphology on skid-resistance of single-grade micro-surfacing. A comparison between single-grade micro-surfacing and MS-2 (which will be explained in Section 2.1.3) standard micro-surfacing was conducted for exploring the feasibility of single-grade micro-surfacing. This study also aimed to correlate conventional texture characteristics with texture results detected by image method.

## 2. Materials and Research Methodologies

The specific contents of this paper are as follows:

1. Three types of fine aggregate are chosen which are steel slag, basalt, and limestone. Three grades 0.6–1.18, 1.18–2.36, and 2.36–4.75 are selected for each type of aggregate.
2. AIMS was used to evaluate morphological characteristics of nine (3 × 3) kinds of fine aggregates.
3. Modified emulsified asphalt used in micro-surfacing was prepared.
4. Nine kinds of micro-surfacing slurry mixture with single-grade aggregate was prepared, corresponding to nine kinds of aggregate. Another normal-grade micro-surfacing mixture was also prepared for comparison with single-grade micro-surfacing. There are, in total, 10 kinds of micro-surfacing in this study.
5. Sand patch test, British pendulum test, and image method were used to evaluate the skid-resistance of micro-surfacing. Wet abrasion test was used to test durability for verifying feasibility of single-grade micro-surfacing.

### 2.1. Materials

#### 2.1.1. Aggregates

Basalt, limestone, and steel slag were selected as fine aggregates of micro-surfacing. Basalt is a basic volcanic rock, with a large compressive strength, low water absorption, and good wear resistance. Limestone has satisfactory physical properties, such as good workability, excellent sturdiness, and good cementation. However, it is generally not used on the surface of asphalt pavement, due to its lower strength than volcanic rocks, such as basalt [25,26]. Steel slag, as an industrial waste, has been more and more widely used in road engineering in recent years [27]. The fine aggregates used in this study were nine types in total: 0.6–1.18, 1.18–2.36, and 2.36–4.75; three grades for each type of aggregate. The basic performance indicators of fine aggregates are shown in Table 1.

#### 2.1.2. Modified Emulsified Asphalt

Modified emulsified asphalt uses asphalt as base material, macromolecule polymer as modifier, and emulsifier added to make asphalt droplets, dissolved in water. It is the binding material of micro-surfacing, and its quality has direct impact on the micro-surfacing’s performance. In order to meet the requirements of rapid open traffic, the emulsifier used in the micro-surface must be slow-breaking and quick-curing cationic emulsifier. The commonly used modifiers are styrene–butadiene–styrene (SBS), styrene–butadiene rubber (SBR), and chloroprene rubber (CR) [32].

The asphalt used in this study was AH-70, where AH stands for heavy traffic paving asphalt, and 70 (unit is 0.1 mm) stands for its penetration. This kind of asphalt has moderate viscoelastic properties and good adhesion properties with aggregates. The modifier was styrene–butadiene rubber (SBR), and the emulsifier is the type of quick-set cationic emulsifier produced by Zhengtong Technology Company. Calcium chloride and polyvinyl alcohol (PVA) are chosen as additives to improve the stability of modified emulsified asphalt, preventing it from quick demulsification during mixing with aggregate.

Colloid mill was used to produce modified emulsified asphalt in this study. The basic principle of colloid mill is that the raw materials, such as asphalt, emulsifier, and water, pass through the gap between the fixed gear and the moving gear, which proceeds at high-speed relative motion, so that the material is subjected to powerful shearing force, friction force, and high-frequency vibration, making it effectively crushed, emulsified, and mixed.

The production process is that stabilizer, emulsifier, and SBR were sequentially added to the water, and mixed uniformly at 65 °C. After adjusting the pH to 2.5, it is put into colloid mill together with hot asphalt to make a modified emulsified asphalt. The proportion of raw materials and basic characteristics of modified emulsified asphalt are shown in Table 2 and Table 3.

#### 2.1.3. Micro-Surfacing Mixture

**Figure 1:**
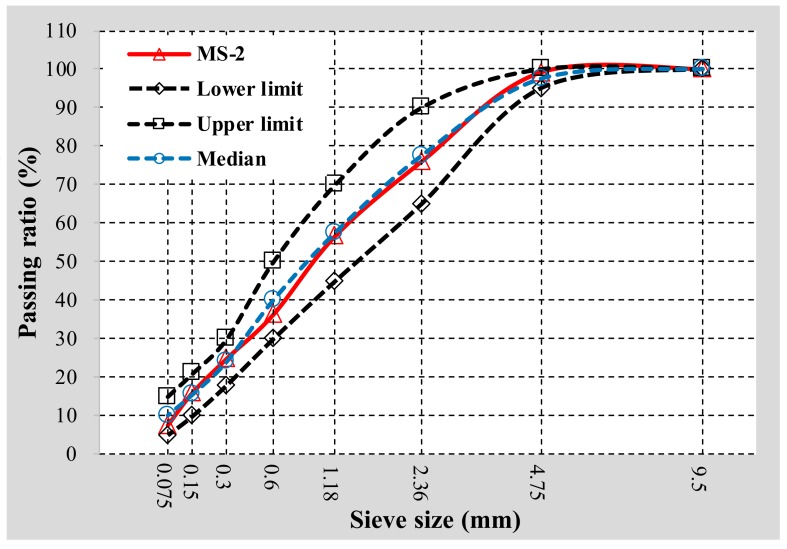
Gradation curves of MS-2 used in this work.

In order to obtain larger texture depth and skid-resistance than ordinary micro-surfacing, this study attempts to prepare single-grade micro-surfacing, which means aggregates in the micro-surfacing mixture have only one size. Corresponding to nine kinds of fine aggregate, nine kinds of single-grade micro-surfacing mixture were prepared. At the same time, in order to verify the feasibility of single-grade micro-surfacing, standard MS-2 micro-surfacing made with basalt was also designed for performance comparison with single-grade micro-surfacing. The grading curve of MS-2 mixture is shown in Figure 1. The proportion of materials in the 10 types of micro-surfacing mix are shown in Table 4. The percentage of cement and water in the table is with regard to the mass of aggregates as denominator.

**Figure 2:**
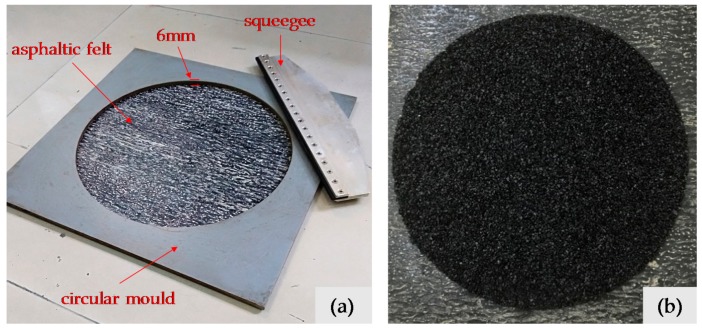
Instruments (**a**) for preparing micro-surfacing specimens (**b**).

The preparation procedures of micro-surfacing specimens used in this study are as follows: (1) Mix a certain amount of aggregate and cement, and then add water and modified emulsified asphalt in proportion according to Table 4 and mixed for one minute; (2) Flatten asphaltic felt and place a circular mold on it. Pour the flowing micro-surface mixture into the 6 mm height circular hole of the mold; (3) Scrape the excess mixture on template surface with a squeegee; (4) Take away the template and put specimens in oven at 60 °C until it reaches constant weight. The instruments for preparation and micro-surfacing specimens are shown in Figure 2.

### 2.2. Research Methodologies

#### 2.2.1. Aggregates Morphology Test

The behaviors of asphalt pavement are directly affected by morphological characteristics of aggregate, which have been demonstrated by many researchers [39,40,41,42,43]. Most traditional test methods of aggregate morphology are indirect and subjective, while AIMS can obtain accurate aggregate morphology information, such as sphericity, angularity, flat and elongated ratio, and texture for coarse aggregate, angularity, and Form2D for fine aggregates through digital image processing.

**Figure 3:**
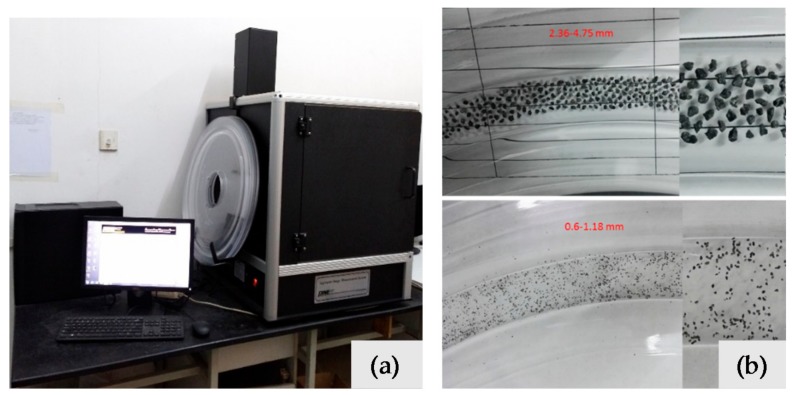
Aggregate image measurement system (**a**) and the tested fine aggregates placed on a tray (**b**).

AIMS consists of image capture hardware and software downloaded on a computer to operate the system and analyze information, which is shown in Figure 3. The image acquisition hardware consists of camera, microscope, aggregate tray, top-lighting, and backlighting systems. The image capture hardware obtains images and detects aggregate by a digital camera and a variable magnification microscope. A profile image of the aggregate is generated by backlighting, from which angularity gradients of the edges are evaluated. Then, top-lighting and variable magnification are utilized to capture surface texture, and test the height of each aggregate particle.

#### 2.2.2. Micro-Surfacing Skid-Resistance Test

Sand patch test [44], British pendulum test [45], and AIMS surface scanning were used to evaluate the texture characteristics in this study. British pendulum test evaluates surface texture of pavement using British pendulum number (BPN). The pendulum, which contains a standard rubber slide, is raised to a locked position, and then it is released, allowing the contact with the specimens’ surface. The BPN is represented by the index where the pointer indicated. Although it is used over the world, it presents some critical issues, since it only describes the friction properties at very low speed.

Sand patch test was conducted in order to make up for the lack of BPN. It consists of spreading 25 cm^3^ of standard sand on clean and dry pavement surface, forming a circle. The diameter of the sand circle is measured, and then the texture depth can be calculated using following equation:

(1) $${\mathrm{TD}\;=\;4V/\pi D}^{2},$$

where TD is texture depth, V is volume of standard sand, and D is diameter of sand circle.

In addition to testing the morphological characteristics of aggregates, AIMS can also provide texture characteristics of the surface of a mixture. It uses a camera to test the height of a 150 mm line on the specimen surface. Each specimen was rotated 45° for new line acquisition, resulting in four scan lines of each specimen. The variances of the heights were used to represent the surface texture of micro-surfacing.

#### 2.2.3. Wet Abrasion Test

Due to the use of a single-grade micro-surfacing, verification of its durability is necessary. Wet abrasion test was used to measure skid-resistance durability. Its main steps include recording the specimen’s mass as m_1_, then fixing the specimen in the tray, adding 25 °C water to completely immerse the sample, and insulation for one hour. The tray was raised so that the sample can jack the abrasion head and make the instrument operate for 300 s. The mass of specimens being abraded were recorded as m_2_. The wet abrasion loss can be calculated using the following equation:

(2) $${\mathrm{WTAT}\;=\;(m}_{1}{\;-\;m}_{2})/A,$$

where WTAT is wet abrasion loss, m_1_ and m_2_ are the mass of specimens before and after abrasion, respectively, and A is the abrasion area of the instrument.

## 3. Results and Discussions

### 3.1. Fine Aggregates Morphology Characteristics

**Figure 4:**
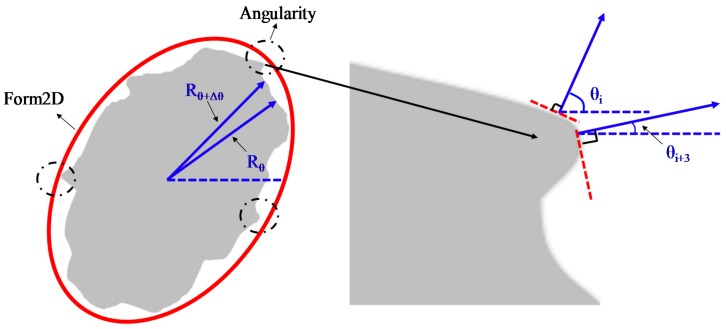
Illustration of Form2D and angularity.

AIMS can evaluate angularity and Form2D for fine aggregate based on digital images. Angularity indicates the edge sharpness of aggregate particles. As the aggregate boundary features change, the angularity of aggregate also changes. AIMS uses the numbers 0–10,000 to express the angularity of each particle. The larger the angularity of aggregate, the sharper the edge of the aggregate is. The value of angularity is calculated based on the gradient on the particle boundary. The gradient technique is operated by calculating the inclination of gradient vectors on particle boundary points from the *x*-axis (horizontal axis in an image). The average change in the inclination of the gradient vectors is taken as an indication of angularity shown in equation (3).

(3) $$\mathrm{Angularity}\;=\;1/(\frac{n}{3}\;-\;1)\overset{n-3}{\underset{i=1}{\sum }}\left|\theta_{i}{\;-\;\theta }_{i+3}\right|,$$

where θ is angle of orientation of the edge points, n is the total number of points, and i is the ith point on the edge of the particle, as shown in Figure 4.

Form2D quantifies the relative form from 2-dimensional images of aggregate particles. Form2D has a relative scale of 0 to 20. A perfect circle has a 2D value of zero. The form index of Form2D is expressed by Equation (4).

(4) $$\mathrm{Form}2D=\overset{\theta =360-\Delta \theta }{\underset{\theta =0}{\sum }}[\frac{R_{\theta +\Delta \theta }-R_{\theta }}{R_{\theta }}],$$

where R_θ_ is the radius of particle at an angle of θ, and Δθ is the incremental difference in the angle. Illustration of Form2D and angularity is shown in Figure 4.

Table 5 summarizes the morphological properties results of fine aggregates. AIMS provides the individual results for each particle analyzed, so the result shown in Table 5 is average values of 200 particles.

According to Table 5, the angularity and Form2D of steel slag are the largest among the three kinds of aggregate, which indicates that the used steel slag in this research has the most abundant morphology characteristics, followed by basalt. Limestone has the closest shape to circle, and the edges of the particles are the smoothest. For different sizes of the same kind of aggregate, angularity and Form2D of 2.36–4.75 grade are the largest of the three sizes of steel slag. For basalt and limestone, aggregate size with the largest angularity is 0.6–1.18, and with the largest, Form2D, is 1.18–2.36. The morphology of aggregates mainly depends on the method of mining and crushing, and there is no specific relationship with the aggregate type and size.

### 3.2. Skid-Resistance and Feasibility of Micro-Surfacing

**Figure 5:**
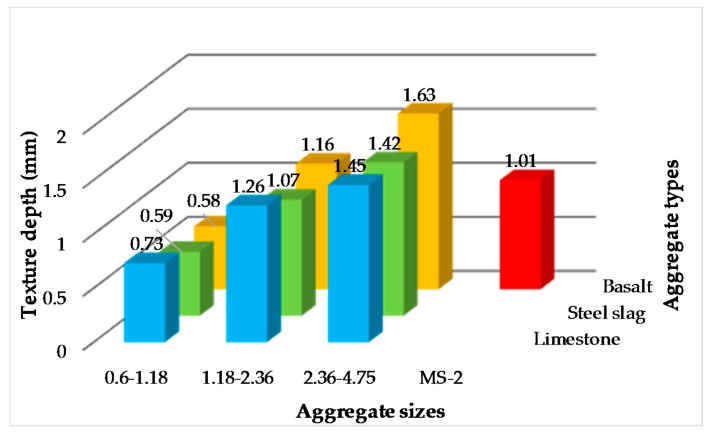
Texture depths of micro-surfacing.

Texture depths of 10 kinds of micro-surfacing evaluated by sand patch test are shown in Figure 5. Firstly, the texture depths of micro-surfacing mixture become larger as the aggregate particle sizes increases when the aggregate source is same. The reason is that the larger the average size of the aggregate particles, the more voids on the surface of the mixture, so that the standard sand can more easily enter the tiny gaps on the surface forming a smaller sand circle. Secondly, the texture depths of 1.18–2.36 and 2.36–4.75 grade micro-surfacing are both greater than those of standard MS-2. It is demonstrated that single-grade micro-surfacing can indeed increase the surface texture characteristic, consequently increasing the skid-resistance.

**Figure 6:**
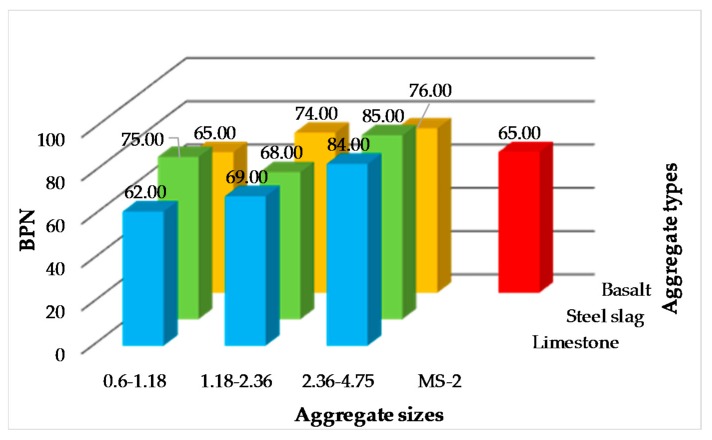
BPN of micro-surfacing.

BPN of 10 kinds of micro-surfacing evaluated by British pendulum test are shown in Figure 6. Firstly, the BPN of micro-surfacing mixture become larger as the aggregate particle sizes increases when the aggregate source is the same, except 0.6–1.18 of steel slag. Secondly, the BPN of micro-surfacing are all larger than those of standard MS-2 except 0.6–1.18 of limestone and basalt. The results also indicated that single-grade micro-surfacing can enlarge the skid-resistance of micro-surfacing.

**Figure 7:**
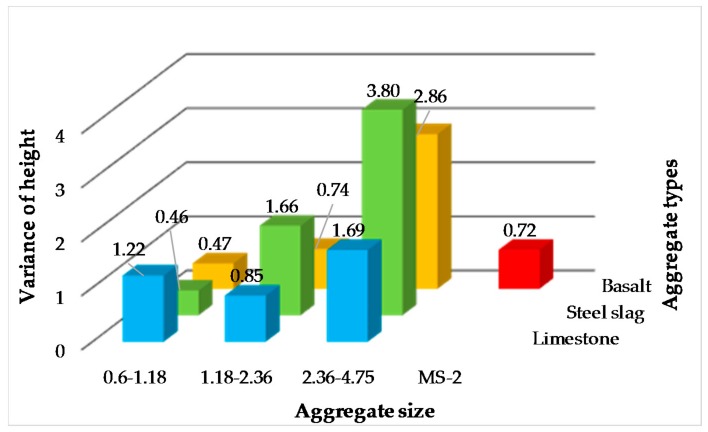
Height’s variance of micro-surfacing.

The variances of heights detected by AIMS were also used to represent the surface texture of micro-surfacing, as shown in Figure 7. Same as the results obtained by the two previous conventional methods, only the 0.6–1.18 of steel slag and basalt micro-surfacing are smoother than standard MS-2. From the results of three kinds of surface texture tests, it can be seen that the surface textures of 1.18–2.36 and 2.36–4.75 single-grade micro-surfacing are larger than MS-2, regardless of the aggregate sources. Therefore, from the perspective of skid-resistance performance, it is feasible to use the 1.18–2.36 and 2.36–4.75 grade aggregates to prepare single-grade micro-surfacing.

**Figure 8:**
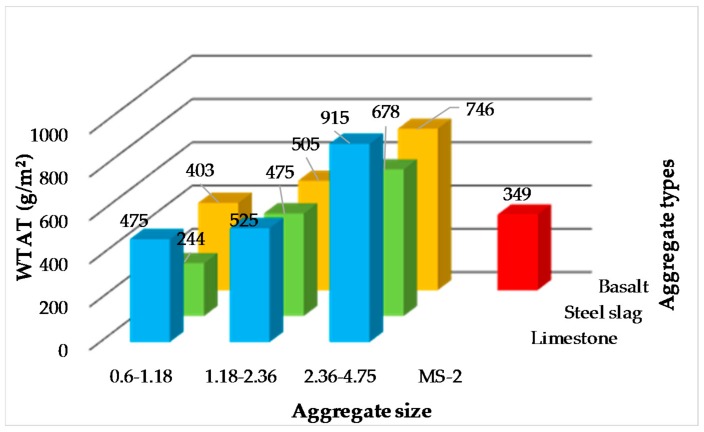
WTAT of micro-surfacing.

Since there is only one size of aggregate in single-grade micro-surfacing, it will lead to the loss of skeleton-embedded structure in mixture, which will then have a certain impact on the strength of micro-surfacing. Therefore, the verification of single-grade micro-surfacing’s durability is necessary. The WTAT of micro-surfacing are shown in Figure 8. The requirement of WTAT is less than 540 in specification. It can be seen that the 0.6–1.18 and 1.18–2.36 grade micro-surfacing meet the requirements. Therefore, 0.6–1.18 and 1.18–2.36 grade micro-surfacing is feasible from the perspective of durability. Combining the feasibility analysis of durability and skid-resistance, it can be seen that the 1.18–2.36 type micro-surfacing meets the requirements of durability and skid-resistance at the same time.

### 3.3. Correlation between Aggregate Morphology and Skid-Resistance

**Figure 9:**
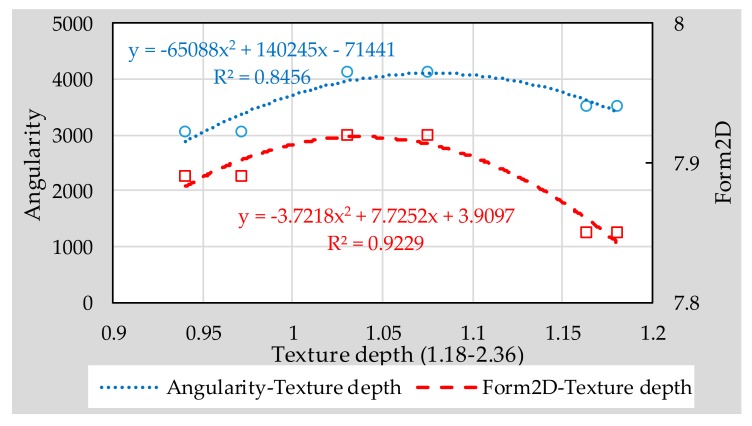
Correlation between aggregate morphology and texture depth.

**Figure 10:**
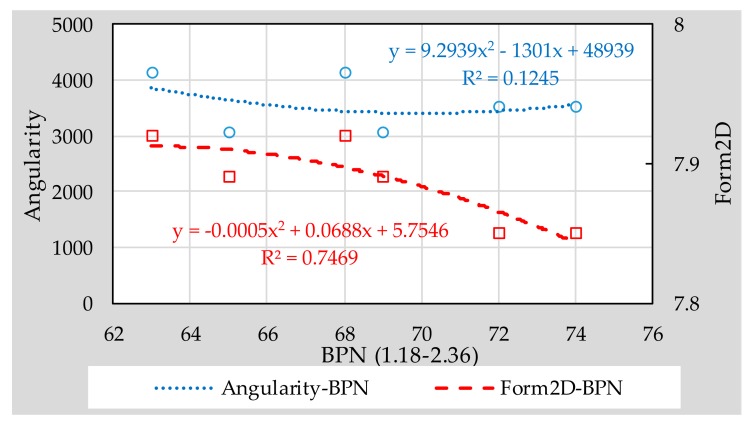
Correlation between aggregate morphology and British pendulum number (BPN).

**Figure 11:**
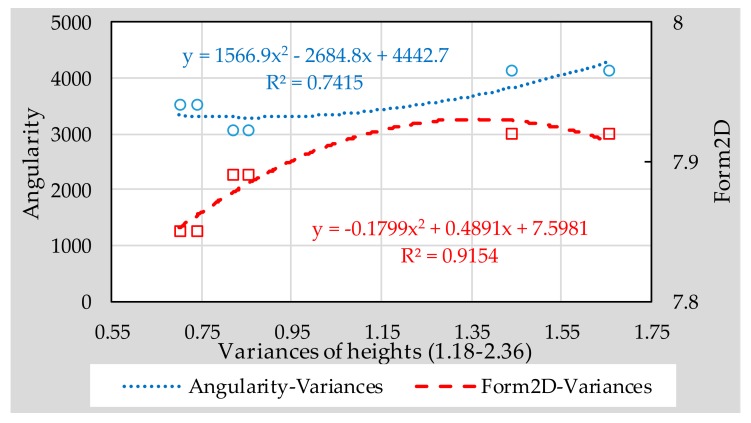
Correlation between aggregate morphology and variances of heights.

From the contents of previous chapter, it can be seen that 1.18–2.36 type micro-surfacing meets the feasibility requirements. Therefore, in this section, the influence of the fine aggregates’ morphological characteristics on the surface texture of 1.18–2.36 micro-surfacing was discussed. The effect of aggregate morphology on texture depth, BPN and variance of height are shown in Figure 9, Figure 10 and Figure 11, respectively. 

It can be seen from Figure 9, Figure 10 and Figure 11 that the *R*^2^ values of the relationship between Form2D and skid-resistance are all larger than those of angularity. This shows that the effect of angularity on the skid-resistance of micro-surfacing is weaker than that of Form2D. The reason might be that the angularity indicates the edge sharpness of aggregate, and the edge sharpness of fine aggregates is too small compared to the surface texture of micro-surfacing, so that there is no obvious relationship between them.

Compared to the BPN, Form2D correlated better with height of variance and texture depth, and the *R*^2^ values reached 0.9154 and 0.9229, respectively. The reason might be that pendulum head’s sliding length of the instrument is difficult to control precisely, resulting in a different friction distance between each specimen, thereby affecting the BPN results. So accurate quantification of texture characteristics should be considered for inclusion in the specification for fully understanding the skid-resistance of pavement.

### 3.4. Correlation between Traditional Test and Digital Image Method

**Figure 12:**
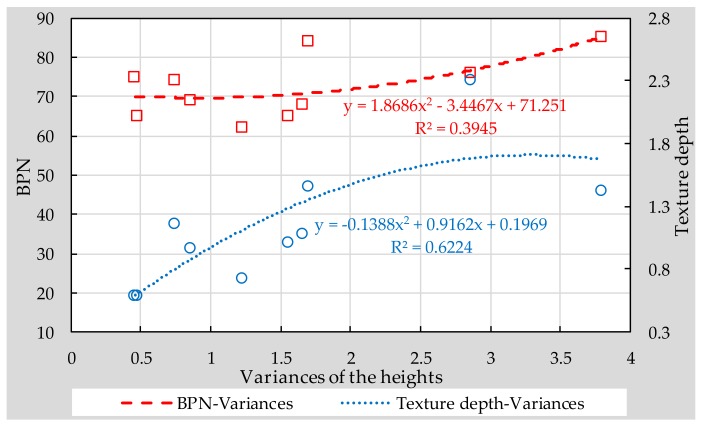
Correlation between traditional test and digital image method for skid-resistance.

Correlations between the traditional test and digital image method for skid resistance were analyzed, as shown in Figure 12. It can be seen that the texture results obtained by sand patch test fit better with the results of image method, compared with British pendulum test. However, the results of *R*^2^ are only 0.6224 and 0.3945 for sand patch test and British pendulum test, respectively, indicating that the proposed quadratic functions between traditional skid-resistance results and image method results are not strong. More data is therefore needed to finalize a more accuracy correlation between traditional test and digital image method, regarding characterization of the texture depth of micro-surfacing.

## 4. Conclusions

The main objective of this study is to explore the effect of aggregate morphology on skid-resistance of single-grade micro-surfacing made with basalt, limestone, and steel slag. A comparison between single-grade micro-surfacing made with three kinds of aggregate and MS-2 standard micro-surfacing consists of basalt was firstly conducted for exploring the feasibility of single-grade micro-surfacing. Then, the correlation between conventional texture characteristics and texture results detected by image method was also discussed. Based on the above results, the following conclusions can be drawn:

1. Single-grade micro-surfacing can indeed increase the surface texture characteristics compared with standard micro-surfacing, thus increasing the skid-resistance.
2. Combining the feasibility analysis of durability and skid-resistance, it can be summarized that the 1.18–2.36 type micro-surfacing meets the requirements of durability and skid-resistance at the same time.
3. Form2D and angularity were characterized as morphologic indices of fine aggregates. Research results indicated that the effect of Form2D on skid-resistance of micro-surfacing is much stronger than that of angularity. Compared with BPN results, height of variance and texture depth correlated better with Form2D.
4. The texture result obtained by sand patch test fits better with results of image method compared with British pendulum test. However, both of them are not strong.

## Figures and Tables

**Table 1:** Fine aggregate basic characteristics.

Properties	Unit	Technical Requirements	Tested Value			Specification Used
			Basalt	Limestone	Steel Slag	
Sand equivalent	%	≥60	68	71	75	ASTM D 2419 [28]
Soundness	%	≥12	15.7	15.1	13.6	ASTM C 88 [29]
Apparent relative density	—	≥2.5	2.978	2.708	2.872	ASTM C 128 [30]
Angularity	s	≥30	42	45	43	ASTM C 1252 [31]

**Table 2:** Composition of modified emulsified asphalt.

Materials	Modified Emulsified Asphalt	Asphalt	Water	SBR	Emulsifier	Calcium Chloride	PVA
Weight (g)	500	300	177	12	10	0.5	0.5
Ratio (%)	100	60	35.4	2.4	2	0.1	0.1

**Table 3:** Basic characteristics of modified emulsified asphalt.

Properties		Unit	Technical Requirements	Tested Value	Specification Used
Sieve residue (1.18)		%	≤0.1	0.04	ASTM D 244 [33]
Charge		—	+	+	ASTM D 244 [33]
Rotary viscosity		Pa · s	—	98	ASTM D 4402 [34]
Evaporated residue content		%	≥60	62.5	ASTM D 244 [33]
Evaporated residue properties	Penetration	0.1 mm	40–100	57.3	ASTM D 5 [35]
	Softening point	°C	≥53	54.8	ASTM D 36 [36]
	Ductility	cm	≥20	56	ASTM D 113 [37]
	Solubility	%	≥97.5	99.3	ASTM D 2042 [38]
Storage stability	1 day	%	≤1	0.1	ASTM D 244 [33]
	5 day	%	≤5	1.2	

**Table 4:** Composition of 10 types of micro-surfacing.

Mixture Types	Limestone			Basalt			Steel Slag			MS-2
	0.6–1.18	1.18–2.36	2.36–4.75	0.6–1.18	1.18–2.36	2.36–4.75	0.6–1.18	1.18–2.36	2.36–4.75	
Thickness (mm)	6	6	6	6	6	6	6	6	6	6
Cement (%)	1	1	1	1	1	1	1	1	1	1
Water (%)	9.5	8.5	7.5	9.5	8.5	7.5	9.5	8.5	7.5	10
Asphalt-aggregate ratio (%)	7.5	7	6.5	7.5	7	6.5	7.5	7	6.5	10

**Table 5:** Morphological properties of fine aggregates.

Properties	Limestone			Basalt			Steel Slag		
	0.6–1.18	1.18–2.36	2.36–4.75	0.6–1.18	1.18–2.36	2.36–4.75	0.6–1.18	1.18–2.36	2.36–4.75
Angularity	3285	3050	2923	3706	3502	3327	3789	4124	4546
Form2D	7.35	7.89	7.20	7.71	7.85	7.64	7.69	7.92	8.33
